# Supplementary material for: Prediction of the Presence of Targetable Molecular Alteration(s) with Clinico-Metabolic 18 F-FDG PET Radiomics in Non-Asian Lung Adenocarcinoma Patients
Source: Diagnostics (Basel). 2022 Oct 10;12(10):2448. doi: 10.3390/diagnostics12102448 (PMC9601118; doi:10.3390/diagnostics12102448)
Supplement: Supplementary file 1 [file diagnostics-12-02448-s001.zip › diagnostics-1938670-supplementary/diagnostics-1938670-supplementary/diagnostics-1938670-supplementary-table s1-done.pdf]

diagnostics

Table S1. Comparison of TrueV and Vereos positron emission tomography quantitative variables before and after the ComBat harmonization process.

Variable	Minimum	Maximum	Mean	P value*	Minimum	Maximum	Mean	P value*
[redacted]								
SUVmean | TrueV	1.316	14.222	4.599					
SUVmean | Vereos	1.565	14.444	5.870	0.001				
SUVmax | TrueV	4.473	35.700	14.717					
SUVmax | Vereos	3.451	32.464	14.046	0.821				
Volume (mL) | TrueV	2.120	615.990	73.234					
Volume (mL) | Vereos	1.008	874.760	99.028	0.988				
TLG (mL)| TrueV	4.132	2448.241	354.571					
TLG (mL)| Vereos	6.709	5722.820	578.423	0.473				
[redacted]								
Skewness | TrueV	-0.077	4.440	0.953					
Skewness | Vereos	-0.251	2.568	0.571	0.006				
Kurtosis | TrueV	1.800	26.763	4.425					
Kurtosis | Vereos	1.925	11.071	3.180	0.032				
ExcessKurtosis | TrueV	-1.200	23.763	1.581					
ExcessKurtosis | Vereos	-1.370	13.516	0.968	0.189				
Entropy_log2 | TrueV	2.145	5.782	4.058					
Entropy_log2 | Vereos	2.259	5.582	4.048	0.978				
Uniformity | TrueV	0.020	0.313	0.089					
Uniformity | Vereos	0.023	0.233	0.079	0.844				
[redacted]								
Sphericity | TrueV	0.716	1.191	0.946					
Sphericity | Vereos	0.649	1.077	0.930	0.670				
Compacity | TrueV	0.735	8.699	2.401		0.492	7.707	3.058	
Compacity | Vereos	1.311	11.225	4.439	**<0.0001**	1.027	7.727	3.048	0.963
[redacted]								
Inverse difference | TrueV	0.202	0.659	0.410					
Inverse difference | Vereos	0.227	0.690	0.464	0.011				
Angular second moment | TrueV	0.001	0.136	0.019					
Angular second moment| Vereos	0.002	0.077	0.016	0.983				
Variance | TrueV	2.169	207.117	28.928		0.383	145.670	19.352	
Variance | Vereos	1.019	60.837	10.932	**<0.0001**	-4.923	141.562	19.352	0.854

Correlation | TrueV	0.211	0.789	0.651		0.278	0.861	0.721	
Correlation | Vereos	0.356	0.945	0.784	**<0.0001**	0.305	0.879	0.721	0.892
Joint entropy | TrueV	3.886	10.681	7.224					
Joint entropy | Vereos	4.105	9.615	7.106	0.674				
Dissimilarity | TrueV	0.952	10.475	3.422		0.839	8.457	2.815	
Dissimilarity | Vereos	0.715	6.411	2.281	**0.0002**	0.685	8.429	2.815	
[redacted]								
Coarseness | TrueV	0.001	0.070	0.018		-0.001	0.057	0.014	
Coarseness | Vereos	0.000	0.054	0.009	**0.001**	0.002	0.070	0.014	0.674
Contrast | TrueV	0.014	0.878	0.198		0.000	0.664	0.141	
Contrast | Vereos	0.008	0.618	0.091	**<0.0001**	0.014	0.942	0.141	0.774
Busyness | TrueV	0.089	4.695	0.838					
Busyness | Vereos	0.066	21.422	1.981	0.007				
[redacted]								
SZE | TrueV	0.229	0.775	0.536		0.211	0.723	0.499	
SZE | Vereos	0.230	0.704	0.466	**0.0004**	0.252	0.748	0.499	0.802
LZE | TrueV	4.368	18386.635	1127.261		-13631.716	635105.336	25996.822	
LZE | Vereos	3.600	1191735.375	47864.883	**<0.0001**	-8620.105	853331.114	25996.822	0.0002
LGZE | TrueV	0.004	0.225	0.034		0.002	0.166	0.024	
LGZE | Vereos	0.001	0.087	0.016	**<0.0001**	-0.001	0.148	0.024	0.631
HGZE | TrueV	36.250	1153.828	228.542					
HGZE | Vereos	18.063	945.575	245.605	0.251				
SZLGE | TrueV	0.003	0.103	0.013		0.002	0.076	0.010	
SZLGE | Vereos	0.001	0.045	0.007	**<0.0001**	-0.001	0.080	0.010	0.353
SZHGE | TrueV	15.577	820.134	148.342					
SZHGE | Vereos	3.238	618.719	126.996	0.433				
LZLGE | TrueV	0.048	704.938	51.188					
LZLGE | Vereos	0.004	28114.797	1269.939	0.296				
LZHGE | TrueV	666.714	1258030.400	50296.736		-155560.617	43339050.699	1561236.634	
LZHGE | Vereos	1793.265	55736935.663	2889821.717	**<0.0001**	-527621.307	39784583.618	1561236.634	**<0.0001**
GLNU | TrueV	1.500	97.667	18.989					
GLNU | Vereos	1.444	607.485	47.394	0.007				

ZLNU | TrueV	2.500	1365.995	150.603					
ZLNU | Vereos	1.222	3926.758	298.169	0.405				
ZP | TrueV	0.033	0.616	0.255		-0.014	0.537	0.196	
ZP | Vereos	0.007	0.625	0.144	**<0.0001**	0.054	0.695	0.196	0.839

* according to Bonferroni correction. a p value < 0.0016 was considered significant.
