# Supplementary material for: Prediction of the Presence of Targetable Molecular Alteration(s) with Clinico-Metabolic 18 F-FDG PET Radiomics in Non-Asian Lung Adenocarcinoma Patients
Source: Diagnostics (Basel). 2022 Oct 10;12(10):2448. doi: 10.3390/diagnostics12102448 (PMC9601118; doi:10.3390/diagnostics12102448)
Supplement: Supplementary file 1 [file diagnostics-12-02448-s001.zip › diagnostics-1938670-supplementary/diagnostics-1938670-supplementary/diagnostics-1938670-supplementary-table s2-done.pdf]

diagnostics

Table S2. LASSO regression coefficients. Variables with null coefficients are not displayed.

	LASSO coefficients
X.Intercept.	*0.188*
Sphericity	-0.293
Sex	-0.234
Tabac	-0.151
AJCC stage	-0.025
GLNU_$_{GLZLM}$	-0.002
Age	0.011
ExcessKurtosis_$_{HISTO}$	0.023
Correlation_$_{GLCM}$	0.503
